# Investigator Use of Social Media for Recruitment of Patients for Cancer Clinical Trials

**DOI:** 10.1001/jamanetworkopen.2020.31202

**Published:** 2020-12-28

**Authors:** Mina S. Sedrak, Can-Lan Sun, Dawn L. Hershman, Joseph M. Unger, Jennifer Liu, William Dale, Don S. Dizon

**Affiliations:** 1 Department of Medical Oncology and Therapeutics Research, City of Hope Comprehensive Cancer Center, Duarte, California; 2 Division of Hematology/Oncology, Department of Medicine, Columbia University Medical Center, New York, New York; 3 Public Health Sciences Division, Southwest Oncology Group Statistics and Data Management Center, Fred Hutchinson Cancer Research Center, Seattle, Washington; 4 Department of Supportive Care Medicine, City of Hope Comprehensive Care Center, Duarte, California; 5 Department of Medical Oncology, Alpert Medical School, Brown University, Providence, Rhode Island; 6 Lifespan Cancer Institute, Rhode Island Hospital, Providence

## Abstract

This survey study discusses the use of social media for recruitment of patients for cancer clinical trials and investigators’ concerns about this recruitment approach.

## Introduction

The use of social media has been explored as a potential strategy to improve enrollment in cancer clinical trials. However, significant barriers to implementation of this recruitment approach remain, including investigator reluctance to use social media for patient recruitment.^1,2^ We conducted a survey study to assess the prevalence of and attitudes toward the use of social media in clinical trial recruitment among cancer investigators.

## Methods

In the Southwest Oncology Group Cancer Research Network, 1 of 4 adult National Cancer Institute–sponsored clinical trials groups, we surveyed 220 principal or coprincipal investigators of National Cancer Institute–funded clinical protocols between October 21 and December 30, 2019. Eligible participants included investigators of study protocols from January 1, 2013, to October 14, 2019. The survey instrument was constructed on the basis of prior qualitative work,^3^ reviewed by methodologists and clinical experts, and pilot tested (eAppendix 1 in the Supplement). The study followed the American Association of Public Opinion Research (AAPOR) reporting guideline^4^ and was approved by the City of Hope institutional review board. The survey was administered online using Research Electronic Data Capture (REDCap) software. Eligible participants were sent an invitation via email with a brief explanation of the purpose of the research and a URL link for online survey. By following the link, participants were provided with a statement of research on the title page of the survey, which included all the elements of informed consent, prior to commencement of the survey.

We measured the prevalence of social media use for trial recruitment as the proportion of respondents (termed *users*) who reported any use (other than “never”) to the item, “How often do you use social media to recruit for your research studies?” We measured attitudes and concerns by dichotomizing responses to survey items as “agree or strongly agree” vs “neutral, disagree, or strongly disagree” and “not at all concerned or slightly concerned” vs “somewhat, moderately, or extremely concerned.”

Demographic and practice characteristics were summarized using descriptive statistics (Table). For each measure, we calculated proportions and 95% CIs. The χ^2^ and Fisher exact tests were used to compare differences between users and nonusers. Logistic regression was used to examine the association between social media use for recruitment and investigator attitudes adjusted for age and protected research time. Analyses were conducted using SAS, version 9.4 software (SAS Institute Inc). The level of statistical significance was set at 2-sided *P* < .05.

**Table.** Investigator Characteristics by Use of Social Media for Cancer Research Recruitment

Characteristic	Social media for recruitment			*P* value
	Users, No. (%) (n = 20)	Nonusers, No. (%) (n = 72)	Total, No. (%) [95% CI] (N = 92)	
Age at the time of survey, y				
≤45	8 (40)	15 (21)	23 (25) [17-35]	.08
>46	12 (60)	57 (79)	69 (75) [65-83]	
Female	10 (50)	31 (43)	41 (45) [34-55]	.58
Non-Hispanic White	6 (30)	21 (29)	27 (29) [20-40]	.94
Physician	17 (85)	60 (83)	77 (84) [75-91]	>.99^a^
Years in practice				
<10	4 (20)	12 (17)	16 (17) [10-27]	.56^a^
10-19	7 (35)	30 (42)	37 (40) [30-51]	
20-29	7 (35)	16 (22)	23 (25) [17-35]	
≥30	2 (10)	14 (19)	16 (17) [10-27]	
Practice setting				
Academic	16 (80)	59 (82)	75 (82) [72-89]	>.99^a^
Nonacademic	4 (20)	13 (18)	17 (18) [11-28]	
Professional time dedicated to clinical care, %				
<30	8 (40)	24 (33)	32 (35) [25-45]	.58
≥30	12 (60)	48 (67)	60 (65) [55-75]	
Professional time dedicated to research, %				
<30	4 (20)	31 (43)	35 (38) [28-49]	.07
≥30	16 (80)	41 (57)	57 (62) [51-72]	
Frequency of social media use for research recruitment				
Once a week or more	3 (15)	NA	3 (3) [0.1-9]	
Less than once a week	17 (85)	NA	17 (19) [11-28]	
Never	NA	72 (100)	72 (78) [68-86]	
Social media platform used for research recruitment				
Twitter	16 (80)	29 (40)	45 (49) [38-60]	
LinkedIn	12 (60)	30 (42)	42 (46) [35-56]	
Doximity	8 (40)	22 (31)	30 (33) [23-43]	
YouTube	8 (40)	12 (17)	20 (22) [14-32]	

Abbreviation: NA, not applicable.

^a^ Obtained with the use of the Fisher exact test. The level of statistical significance was set at *P* < .05, 2-sided.

## Results

The survey response rate was 45% (92 of 220 surveys were returned) (eAppendix 2 in the Supplement). Twenty of 92 participants (22%; 95% CI, 14%-32%) reported that they use social media platforms to recruit patients (users). Compared with nonusers, users were more likely to be younger (<45 years, 8 of 20 [40%] vs 15 of 72 [21%]; *P* = .08), and had 30% or more professional time dedicated to research (16 of 20 [80%] vs 41 of 72 [57%]; *P* = .07).

The majority of the 92 participants agreed that social media can help increase awareness of cancer trials (79 [86%; 95% CI, 77%-92%]), education about cancer trials (75 [82%; 95% CI, 72%-89%]), and patient access to cancer trials (69 [75%; 95% CI, 65%-83%]) (Figure, A). Most participants were concerned about the risk of misinformation (55 [60%; 95% CI, 49%-70%]) and misinterpretation (53 [58%; 95% CI, 47%-68%]) of the trial information, and 54 participants (59%; 95% CI, 48%-69%) believed that trained moderator monitoring is needed. Compared with users, nonusers were more concerned about misinformation (49 of 72 participants [68%] vs 6 of 20 [30%], *P* = .004), misinterpretation (48 [67%] vs 5 [25%], *P* = .002), undisclosed conflicts of interest (32 [44%] vs 3 [15%], *P* = .02), and violations of patient privacy (27 [38%] vs 1 [5%], *P* = .005) (Figure, B). Adjustment for age and protected research time did not change these associations.

**Figure.**
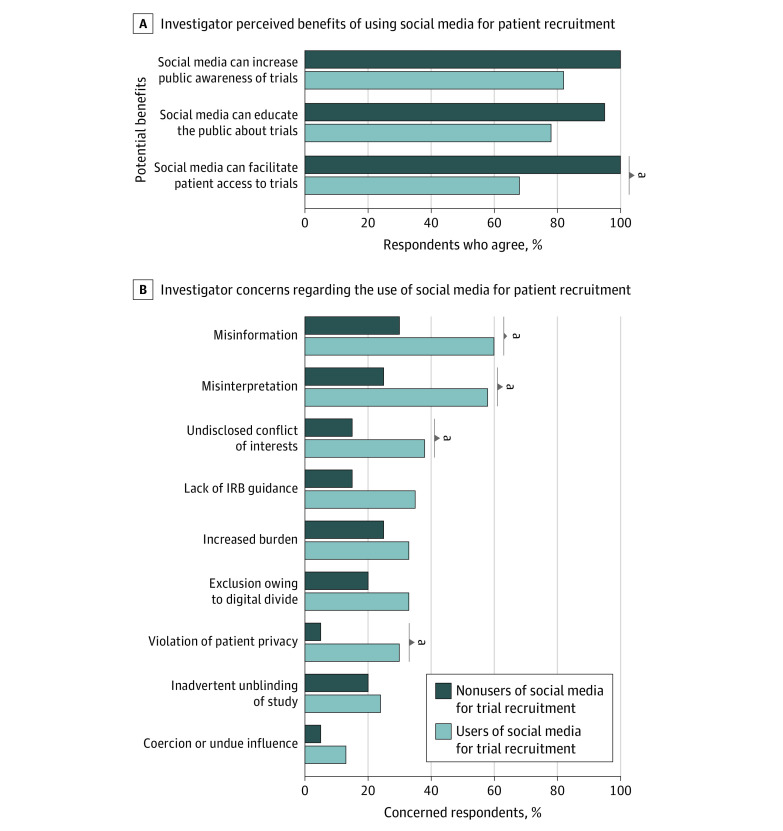
Survey Results A, Proportion of Southwest Oncology Group (SWOG) investigators surveyed who agree on the potential benefits of using social media for patient recruitment. B, Proportion of SWOG investigators surveyed who have the listed concerns regarding the use of social media for patient recruitment. ^a^Indicates a statistically significant difference between users and nonusers at *P* < .05.

## Discussion

Nearly 1 in 4 cancer investigators in the Southwest Oncology Group reported that they used social media to recruit patients for trials. Although most investigators were optimistic about social media use for this purpose, many barriers to use remain. This exploratory study was limited to investigators of a single National Cancer Institute network group.

With more than 2.9 billion individuals worldwide using social media platforms regularly, their use has created an ability to disseminate health information with unprecedented speed, reach, and penetration.^5^ Use of such platforms has become increasingly important amid the coronavirus disease 2019 pandemic,^5^ prompting discussions in the scientific literature and popular press about the potential application of these widely used platforms to improve patient recruitment for trials.^6^ If investigator resistance is overcome, social media may emerge as an essential tool for promoting accrual to clinical trials.

## Notes

Supplement. **eAppendix 1.** Survey Instrument **eAppendix 2.** Response Rate Calculation
