# Errors in Figure

**DOI:** 10.1001/jamanetworkopen.2021.5118

**Published:** 2021-03-18

In the Research Letter titled “Investigator Use of Social Media for Recruitment of Patients for Cancer Clinical Trials,”^1^ published December 28, 2020, errors occurred in the Figure. In the figure’s key, the group identifications should have been reversed: the dark blue color should have indicated the users group, and the light blue color should have indicated the nonusers group. In addition, the absolute percentage values for the nonusers group were slightly off. This article has been corrected.^1^
