# Supplementary material for: Investigator Use of Social Media for Recruitment of Patients for Cancer Clinical Trials
Source: JAMA Netw Open. 2020 Dec 28;3(12):e2031202. doi: 10.1001/jamanetworkopen.2020.31202 (PMC7770556; doi:10.1001/jamanetworkopen.2020.31202)
Supplement: Supplement. — eAppendix 1. Survey Instrument eAppendix 2. Response Rate Calculation [file jamanetwopen-e2031202-s001.pdf]

Supplemental Online Content

Sedrak MS, Sun CL, Hershman DL, et al. Investigator use of social media for recruitment of patients for cancer clinical trials. *JAMA Netw Open.* 2020;3(12):e2031202. doi:10.1001/jamanetworkopen.2020.31202

eAppendix 1. Survey Instrument

eAppendix 2. Response Rate Calculation

This supplemental material has been provided by the authors to give readers additional information about their work.

© 2020 Sedrak MS et al. *JAMA Network Open.*

eAppendix 1. Survey Instrument

Social Media Use in Cancer Research

October 21, 2019

I. Consent

Do you agree to participate in this survey?

Yes
No [Terminate]

II. Social Media Use

We define social media as forms of electronic communication, such as websites for social networking and blogging, through which users share information, ideas, and other content.

1. In the past 6 months, how often did you visit or use the following social media sites for PERSONAL purposes (e.g., sharing life events, photos, and news with close family and friends)?

	Never	Less than every few weeks	Every few weeks	Once a week	A few times a week	About once a day	Several times a day
Twitter							
Facebook							
Instagram							
Snapchat							
LinkedIn							
Doximity							
ResearchGate							
YouTube							
Blogs							

2. In the past 6 months, how often did you visit or use the following social media sites for PROFESSIONAL purposes (e.g., sharing your work with a public audience, fostering new collaborations, and/or connecting with colleagues, students, or patients)?

	Never	Less than every few weeks	Every few weeks	Once a week	A few times a week	About once a day	Several times a day
Twitter							
Facebook							
Instagram							
Snapchat							
LinkedIn							
Doximity							
ResearchGate							
YouTube							
Blogs							

© 2020 Sedrak MS et al. *JAMA Network Open.*

3. Thinking about your use of social media sites… how often do you use social media to…

	Never	Less than every few weeks	Every few weeks	Once a week	A few times a week	About once a day	Several times a day
Network							
Disseminate your research							
Consult colleagues regarding patient issues							
Make referrals							
Share cases and ideas							
Market your practice or organization							
Promote your accomplishments (e.g., grants or awards)							
Communicate directly with patients							
Recruit for your research studies							
Participate in online communities (e.g., Facebook groups or Twitter chats)							
Learn about new research articles							
Read research articles							
Learn about news headlines/ breaking news							
Read news articles							

III. Social Media Use for Clinical Trial Recruitment

4. Please indicate your level of agreement with the following statements:

	Strongly disagree	Disagree	Neutral	Agree	Strongly agree
Social media can increase public awareness of clinical trials.					
Social media can be used to educate the public about clinical trials.					
Social media can facilitate patient access to clinical trials.					

© 2020 Sedrak MS et al. *JAMA Network Open.*

5. I am concerned about using social media in clinical trials due to…

	Not at all concerned	Slightly concerned	Somewhat concerned	Moderately concerned	Extremely concerned
Exclusion of people who have limited online engagement (e.g., older adults)					
Misinformation by patients or other non-experts (e.g., spread of false, inaccurate, or incomplete health information)					
Misinterpretation of trial rationale, design, and/or procedures by patients or other non-experts					
Inadvertent "unblinding" of a study that could lead to increased drop out, altered outcomes					
Increased burden (e.g., investigator time, resources, funding)					
Lack of IRB guidance on safe and effective social media use					
Violation of HIPAA/patient privacy and confidentiality					
Coercion or undue influence					
Promotion of research with undisclosed conflict of interests					

Other concerns: ______________________________

IV. Strategies and Interventions

6. I would use (or increase my use of) social media for clinical trials if there were…

	Strongly disagree	Disagree	Neutral	Agree	Strongly agree
Private social media groups for trial participants					
Trained moderators who vet and closely monitor all communication for sensitivity and potential harms (e.g., patient protection and study integrity)					
Algorithms to identify misinformation (e.g., technocognition)					
Processes to acquire consent to contact potential participants on social media					
Evidence-based frameworks on social media					
Tutorials and educational materials					

© 2020 Sedrak MS et al. *JAMA Network Open.*

V. Misinformation on Social Media

7. To mitigate the spread of misinformation on social media, I believe we need to…

	Strongly disagree	Disagree	Neutral	Agree	Strongly agree
Moderate social media channels via moderators to keep people on track, minimize trolling					
Educate patients about trials and trial design					
Educate users so that they may be able to recognize misinformation (e.g., media literacy)					

8. We invite you to share your thoughts about social media and its use as a research tool. Please use the text box provided.

VI. Demographics

9. Sex:

Male
Female

10. Current age (in years):

<25
25-35
36-45
46-55
56-65
66-75
>75

11. How would you describe your race?

American Indian or Alaska Native
Asian
Black or African American
Native Hawaiian/Other Pacific Islander
White
Other

© 2020 Sedrak MS et al. *JAMA Network Open.*

12. Are you of Hispanic, Latino, or of Spanish origin?

Yes
No

13. Profession:

Physician
Physician Assistant
Nurse Practitioner
Nurse
Pharmacist
Researcher
Other

If you selected “Other,” please specify: ______________________________

14. Medical specialty:

Medical Oncology
Radiation Oncology
Surgical Oncology
Other
Not applicable

15. Years in practice:

<5
5-<10
10-19
20-29
30-39
40-49
>=50

16. State of residence: [drop down selection]

17. Do you currently see patients?

Yes
No

18. What is your primary practice setting?

Solo Practice
Group Practice
Hospital Owned
Federally Qualified Health Center
Academic Health Center
VA
Other

© 2020 Sedrak MS et al. *JAMA Network Open.*

19. Approximately what percentage of your professional time is dedicated to clinical responsibilities/patient care?

< 10%
10-<30%
30-<70%
70-<90%
> =90%

20. Approximately what percentage of your professional time is dedicated to research or teaching?

< 10%
10-<30%
30-<70%
70-<90%
>= 90%

© 2020 Sedrak MS et al. *JAMA Network Open.*

Appendix e2. Response Rate Calculation

We used the AAPOR response rate 3 definition which takes into account the proportion of cases of unknown eligibility that may in fact be eligible ("e").

$$e = \frac{\textit{eligible respondents}}{\textit{total respondents}} = \frac{92}{98} = 93.8\%$$

$$RR3 = \frac{\textit{eligible respondents}}{\textit{eligible respondents} + e(\textit{non-respondents})} = \frac{92}{92 + 0.938(220-98)} = 44.6\%$$

© 2020 Sedrak MS et al. *JAMA Network Open.*
